# Chronic cathepsin inhibition by E‐64 in Dahl salt‐sensitive rats

**DOI:** 10.14814/phy2.12950

**Published:** 2016-09-04

**Authors:** Gregory Blass, Vladislav Levchenko, Daria V. Ilatovskaya, Alexander Staruschenko

**Affiliations:** ^1^ Department of Physiology Medical College of Wisconsin Milwaukee Wisconsin

**Keywords:** Cathepsin, cathepsin L, cysteine proteases, salt‐sensitive hypertension

## Abstract

Cysteine cathepsins are lysosomal enzymes expressed in the kidneys and other tissues, and are involved in the maturation and breakdown of cellular proteins. They have been shown to be integrally involved in the progression of many cardiovascular and renal diseases. The goal of this study was to determine the involvement of cysteine cathepsins in the development of salt‐sensitive hypertension and associated kidney damage. In our experiments, Dahl salt‐sensitive (SS) rats were fed an 8% high salt NaCl diet and intravenously infused with the irreversible cysteine cathepsin inhibitor E‐64 (1 mg/day) or the vehicle (control). Both the control and E‐64 infused groups developed significant hypertension and kidney damage, and no difference of the mean arterial pressure and the hypertension‐associated albuminuria was observed between the groups. We next tested basal calcium levels in the podocytes of both control and infused groups using confocal calcium imaging. Basal calcium did not differ between the groups, indicative of the lack of a protective or aggravating influence by the cathepsin inhibition. The efficacy of E‐64 was tested in Western blotting. Our findings corresponded to the previously reported, E‐64 induced increase in cathepsin B and L abundance. We conclude that the inhibition of cysteine cathepsins by E‐64 does not have any effects on the blood pressure development and kidney damage, at least under the studied conditions of this model of SS hypertension.

## Introduction

Cathepsins (Cath) are proteolytic enzymes that are important in such cellular functions as the breakdown, maturation, and modulation of the bioactivity of proteins. Three major groups of Caths have been categorized: syrine (A and G), aspartic (D and E), and cysteine Caths. Cystein Caths, members of the family of papain‐like cysteine proteases, are the largest Cath family in humans that is comprised of 11 proteases (the Caths B, C, F, H, K, L, O, S, V, X and W) (Turk et al. 2012). These enzymes, which are stable in acidic environments, are primarily found in lysosomes and endosomes. However, active forms and splice variants can be found in the extracellular (Reddy et al. 2001; Jaiswal et al. 2002), cytosolic, mitochondrial (Muntener et al. 2004), and nuclear compartments (Goulet et al. 2004; Alcalay et al. 2008).

Cath B and L are among the most abundant lysosomal proteases (Rossi et al. 2004). Both Cath B and L are endopeptidases, however Cath B contains an occluding loop (Musil et al. 1991) that blocks the active site and prevents endopeptidase activity at low pH levels. At less acidic pH, the loop opens, enabling Cath B endopeptidase activity (Illy et al. 1997). Both Cath B (Kominami et al. 1985) and L (Sever et al. 2007) are abundantly expressed within the kidney and Cath L expression has been shown to be strongly associated with the extent of renal injury (Bauer et al. 2011). How these and other Caths contribute to a variety of diseases has been of recent interest.

Previous studies have shown beneficial in vivo effects of cysteine Cath inhibition in several disease models. These include chronic kidney disease (Bauer et al. 2011; Fox et al. 2016), hypertensive heart failure (Cheng et al. 2008), and polycystic kidney disease (Igarashi and Somlo 2007; Alcalay et al. 2008). However, cysteine Cath inhibition in the context of salt‐sensitive (SS) hypertension has yet to be studied. SS hypertension is characterized by an elevated mean arterial pressure (MAP), associated kidney damage, and proteinuria caused by a high salt (HS) diet. Previous use of Cath inhibitors in disease models suggests that multiple Caths may contribute synergistically to disease progressions and the use of a nonspecific Cath inhibitor may be a more effective approach (Siklos et al. 2015). Thus, E‐64, a nonspecific inhibitor, was used to asses cysteine Cath function in SS hypertension.

The effacement of glomerular podocytes, which are essential for the glomerular filtration barrier (Pavenstadt et al. 2003), can cause proteinuria (Nagase et al. 2006). Previously, the in vitro inhibition of renal cysteine Caths was shown to attenuate this effacement (Sever et al. 2007; Faul et al. 2008). However, whether this observation will extend to the in vivo inhibition and other pathophysiological effects of SS hypertension has yet to be determined. Sustained Ca^2+^ overload in glomerular podocytes was also previously indicated as a driving factor of the podocyte effacement (Lavin and Winn 2011). Therefore, the study here measured E‐64 induced changes of glomerular podocyte basal [Ca^2+^]_i_ in SS rats. Overall, the goal of the study was to identify whether the in vivo inhibition of cysteine Caths attenuates SS hypertension, and the coupled proteinuria and kidney damage.

## Material and Methods

### Animals

Animal use and welfare adhered to the NIH Guide for the Care and Use of Laboratory Animals following a protocol reviewed and approved by the IACUC of the Medical College of Wisconsin. Seven‐week old male Dahl Salt Sensitive rats (SS/JrHsdMcwi) were used. Food (0.4% or 8.0% NaCl AIN‐76 purified rodent food; Dyets, Bethlehem, PA) and water were provided ad libitum. Animals were housed in metabolic cages 7 days prior to femoral artery and vein catheterization. They were then returned to the metabolic cages and allowed to recover from surgery for 5–7 days.

### Chronic instrumentation for venous infusion and blood pressure measurement

Chronic instrumentation of rats for venous infusion and blood pressure measurements was done as described previously. Briefly, 8‐week old anesthetized SS rats had their left femoral artery and vein catheterized. Both catheters were fixed and exteriorized from the back of the neck and the arterial line was connected to a heparinized saline infusion pump that was in line with a blood pressure transducer, and the venous line was connected to a saline infusion pump. Animals were allowed 360° movement using a tether‐swivel system. This preparation allowed chronic venous infusion and arterial blood pressure measurement in conscious, freely moving rats. A stable baseline blood pressure was obtained for 4 days prior to switching both groups to an 8.0% NaCl diet and the simultaneous addition of N‐[N‐(L‐3‐trans‐carboxyox‐irane‐2‐carbonyl)‐L‐leucyl]‐agmatine (E‐64, 1 mg/day; 280 mmol/L stock in DMSO; ApexBio, Houston, TX, Cat #A2576) or the vehicle (DMSO in saline) control to the venous catheter. Daily MAP was calculated by averaging MAP taken every min over the beginning 3 h period of the rat sleep cycle.

### Blood and urine electrolyte measurement

By detaching the femoral arterial catheter from the swivel, 1 mL of arterial blood was collected the morning of the diet change and at every subsequent 7th day. Twenty‐four hour urine was collected with the metabolic cage apparatus on the same day as blood collections. Whole blood and urine electrolytes were measured with a blood gas and electrolyte analyzer (ABL system 800 Flex; Radiometer, Copenhagen, Denmark). Albumin was measured using a fluorescent assay (Albumin Blue 580 dye; Molecular Probes, Eugene, OR), read by a fluorescent plate reader (FL600; Bio‐Tek, Winooski, VT), and creatinine using a Jaffé reaction‐assay using an autoanalyzer (ACE; Alfa Wasserman, West Caldwell, NJ). Inorganic phosphorus was measured by chemical reduction to form a phosphomolybdate complex (P7516; Pointe Scientific, Canton, MI) and read by a fluorescent plate reader (SpectraFluor Plus; Tecan). Ammonia was quantified using an enzymatic reagent kit (A7553; Pointe Scientific) and read by a fluorescent plate reader (SpectraFluor Plus; Tecan, Männedorf, Switzerland).

### Kidney isolation

Rats were anesthetized, and their descending aortas were catheterized. Kidneys were flushed via the catheter with PBS (3 mL/min/kidney) until blanched. Kidneys were excised and decapsulated. Half of the right kidney was used for glomerular podocyte basal [Ca^2+^]_i_ measurements and the other half was used for Western blot analysis. The left kidney was used for histochemistry staining.

### Histochemistry and Western blot analysis

Rat kidneys were fixed in zinc formalin, paraffin embedded, sectioned, and mounted on slides. Slides were stained with the Masson's trichrome stain. Renal cortical protein levels were determined by immunoblotting on unfixed, frozen samples with antibodies for Cath B (sc‐6493; Santa Cruz, Dallas, TX) and Cath L (sc‐6498; Santa Cruz). Kidney cortical sections were isolated and then lysed. Equal amounts of cell lysate were subjected to Western blot analysis with the Cath antibodies and *β*‐actin as was previously described (Karpushev et al. 2011).

### Isolation of glomeruli and measurement of podocyte basal [Ca^2+^]_i_

Isolation and basal podocyte [Ca^2+^]_i_ measurements were done as has been previously reported (Ilatovskaya and Staruschenko 2013; Ilatovskaya et al. 2015b). Briefly, the kidney cortex was mechanically isolated and minced until homogenous. The minced tissue was pushed through a 100 followed by a 140 mesh sieve. The flow‐through was filtered by a 200 mesh sieve and the sieve‐sedimented glomeruli were collected. Isolated glomeruli were incubated with Fura Red, AM and Fluo‐4, AM (5 *μ*mol/L; Invitrogen, Grand Island, NY) for 40 min at room temperature. Glomeruli were adhered to poly‐l‐lysine coated glass coverslips and imaged using a confocal laser‐scanning microscope.

Calcium imaging was performed as has been previously described (Ilatovskaya and Staruschenko 2013; Ilatovskaya et al. 2014, 2015a,b). A laser‐scanning confocal microscope system (Nikon A1‐R, NIS Elements; Nikon Instruments Inc, Tokyo, Japan) was used to collect images in time series (xyt, 4 s per frame). Basal [Ca^2+^]_i_ of podocytes were measured after stable fluorescence intensities were established. Only surface podocytes of glomeruli were recorded. Each experiment measured basal [Ca^2+^]_i_ of 4 to 9 podocytes of at least one glomerulus imaged.

### Statistical analysis

Data are presented as mean ± SEM. The MAP, blood, and urine measurements were compared using the two‐way analysis of variance (ANOVA) for repeated measures followed by the Holm–Sidak post hoc test. For the podocyte calcium measurements, the values of [Ca^2+^]_i_ at every moment of time for individual cells were averaged by the number of regions registered in the experiment. Data for kidney/TBW ratios, podocyte calcium measurements, and the relative Cath protein levels were compared using the independent‐samples *t*‐test. *P* values less than 0.05 were considered significant.

## Results

### Mean arterial pressure of catheterized SS rats

**Figure 1:**
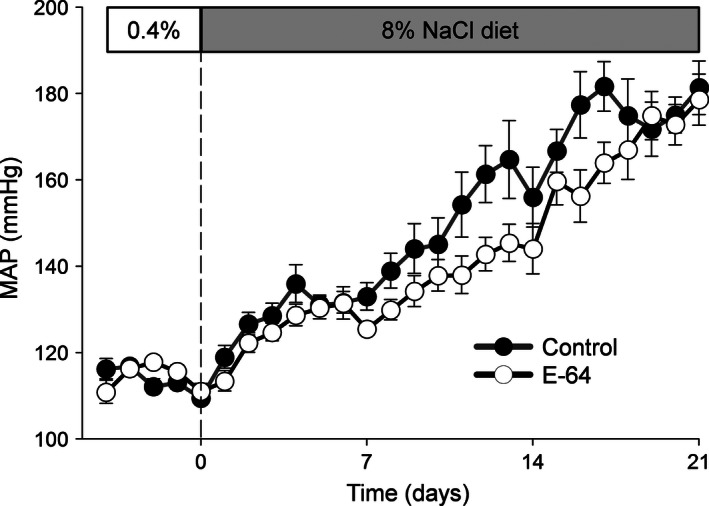
High salt induced hypertension in Dahl salt‐sensitive (SS) rats. Mean arterial blood pressures (MAP) of SS rats were measured during a 0.4% NaCl diet for 4 days followed by 21 days of a 8.0% NaCl diet. Vehicle or E‐64 (1 mg/day) was i.v. infused after switch to HS diet during the entire protocol (Control *N* = 5, E‐64 *N* = 8; error bars, SE).

Basal MAP was recorded for 4 days prior to the switch of diets to 8.0% NaCl and the addition of 1 mg/day of E‐64 to venous infusate. Figure 1 illustrates the typical progressive increase in the rat MAP induced by the 21 day 8.0% NaCl diet (Moreno et al. 2011; Endres et al. 2014; Cowley et al. 2016). The E‐64 treatment did not attenuate MAP (*P *=* *0.239, two‐way ANOVA) and kidney weights were similar between E‐64 treated (*M *=* *1.440 ± 0.051) and the control group (*M *=* *1.454 ± 0.044) (*P *=* *0.857, independent‐sample *t*‐test; data not shown).

### Blood electrolytes

**Figure 2:**
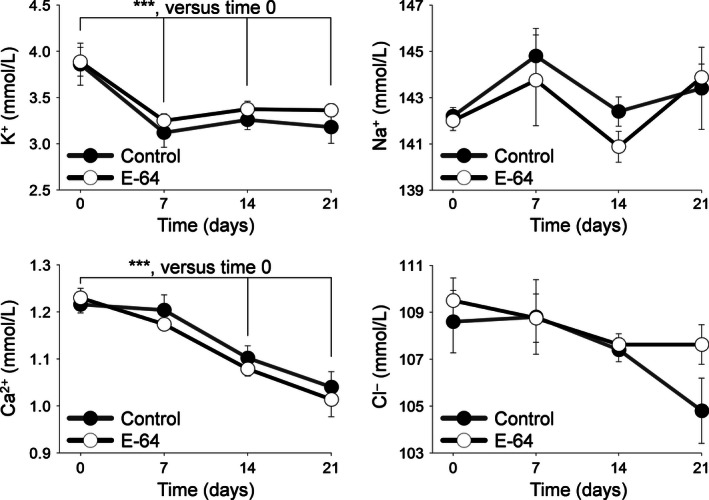
Whole blood electrolytes. Arterial whole blood electrolyte concentrations were measured during 0.4% NaCl diet at day 0 and on an 8.0% NaCl diet at days 7, 14, and 21 for K^+^, Na^+^, Ca^2+^, and Cl^−^. ***indicates a *P* value < 0.001. Two‐way analysis of variance (ANOVA) for repeated measures; Holm‐Sidak post hoc (Control *N* = 5, E‐64 *N* = 8; error bars, SE).

Blood electrolyte concentrations are shown in Figure 2. SS rats fed an 8.0% NaCl diet had significantly reduced blood [K^+^] in the first 7 days on the diet that continued through the remainder of the diet (*P *<* *0.001, two‐way ANOVA). [Ca^2+^] gradually decreased during the 21 day diet (*P* < 0.001, two‐way ANOVA). However, no significant change was observed for [Na^+^] and [Cl^−^]. All measured blood electrolyte concentrations were no different between the two groups.

### Urinary output, electrolytes, and albumin, phosphorus, and ammonia/creatinine ratios

**Figure 3:**
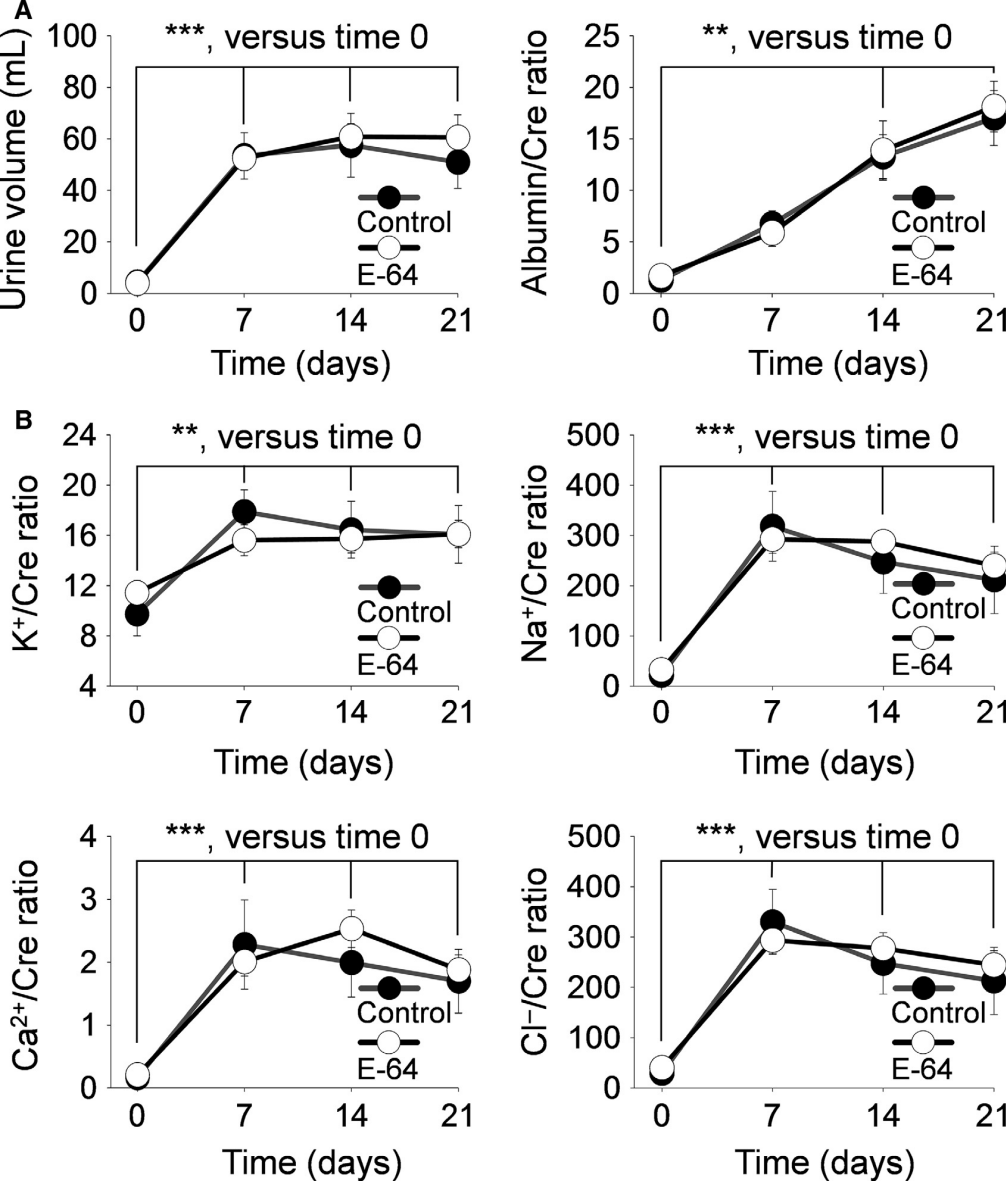
Albumin and electrolyte urinary excretion. Urine was collected during a 0.4% NaCl diet at day 0 and on an 8.0% NaCl diet at days 7, 14, and 21. (A) Left panel, 24 h urine volume; Right panel, albumin/creatinine ratios, (B) urine electrolyte/creatinine ratios for K^+^, Na^+^, Ca^2+^, and Cl^−^. ** and ***indicates a *P* value < 0.01 and 0.001, respectively. Two‐way analysis of variance (ANOVA) for repeated measures; Holm‐Sidak post hoc (Control *N* = 5, E‐64 *N* = 8; error bars, SE).

**Figure 4:**
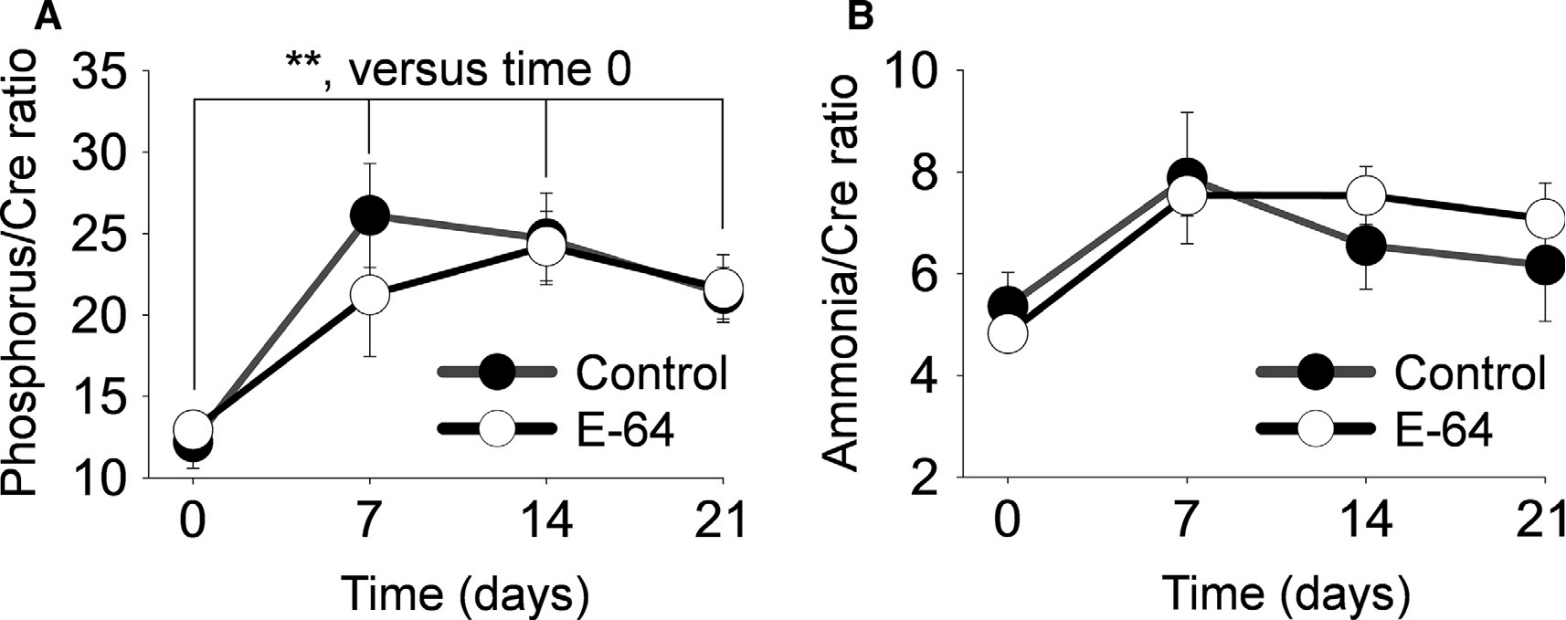
Inorganic phosphorus and ammonia urinary excretion. Urine was collected during a 0.4% NaCl diet at day 0 and on an 8.0% NaCl diet at days 7, 14, and 21. (A) Urine inorganic phosphorus/creatinine ratios. (B) Urine ammonia/creatinine ratios. **indicates a *P* value < 0.01. Two‐way analysis of variance (ANOVA) for repeated measures; Holm‐Sidak post hoc (Control *N* = 5, E‐64 *N* = 8; error bars, SE).

An 8.0% NaCl diet induced a significant increase in the daily urine volume by day 7 that continued for the following 14 days (*P *<* *0.001, two‐way ANOVA), and a steady, significant increase in albuminuria (normalized to creatinine) was observed during the 21 days of the HS diet compared to day 0 (*P *<* *0.01, two‐way ANOVA) (Fig. 3A). Of the urine electrolytes measured (Fig. 3B), K^+^ (*P* < 0.01), Na^+^, Ca^2+^, and Cl^−^ excretion significantly increased after day 0 (normalized to creatinine, *P *<* *0.001, two‐way ANOVA). Urinary phosphorus significantly increased after day 0 (*P* < 0.001, two‐way ANOVA) (Fig. 4A). Urinary ammonia did not significantly increase (Fig. 4B) and no difference was found between the groups of all urine measurements.

### E‐64 effect on glomeruli and podocytes

**Figure 5:**
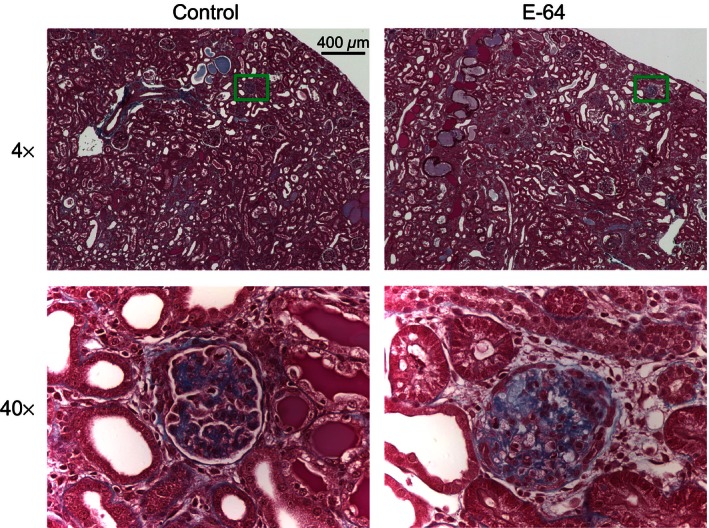
Representative image of trichrome‐stained kidney sections. Left, images are of a 4× (top) and 40× (bottom) of the control rats; Right, images of the E‐64 treated group. Green boxes on 4× images represent locations of the 40× images. Scale bar is shown.

**Figure 6:**
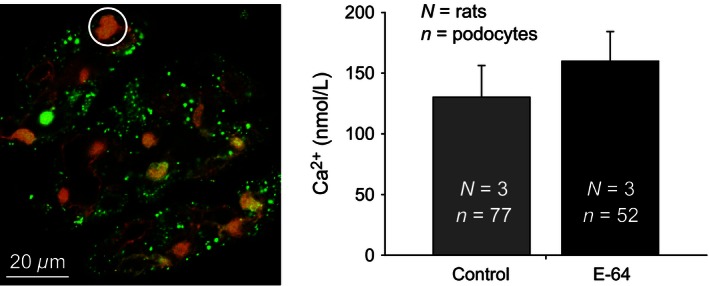
Intracellular basal calcium in the podocytes of freshly isolated glomeruli from E‐64 treated and control rats. Left panel, a representative ratiometric fluorescence image of a rat glomerulus loaded with Fluo‐4 (green pseudocolor) and FuraRed (red pseudocolor) calcium dyes; the white circle identifies an example of a podocyte (ROI) used in the basal [Ca^2+^]_i_ calculations. Right panel summarizes the basal [Ca^2+^]_i_ of glomerular podocytes from control and E‐64 treated rats fed an 8.0% NaCl diet for 21 days (*N* = 3 for both groups; error bars, SE).

Taking into consideration that E‐64 inhibition of Caths has been shown to attenuate glomerular damage, histological coronal sections of the kidneys were done. Figure 5 shows representative renal and glomeruli damage observed in SS rats after the 21 days of an 8.0% NaCl diet. The E‐64 infused rats had no observable change in glomeruli damage. Basal [Ca^2+^]_i_ has been shown to be linked with podocyte dysfunction. Here, we measured basal [Ca^2+^]_i_ of podocytes in freshly isolated glomeruli (Fig. 6). The E‐64 treated rats had no difference in basal podocyte [Ca^2+^]_i_ (*P *=* *0.429, independent‐sample *t*‐test).

### Cathepsin B and L expression

**Figure 7:**
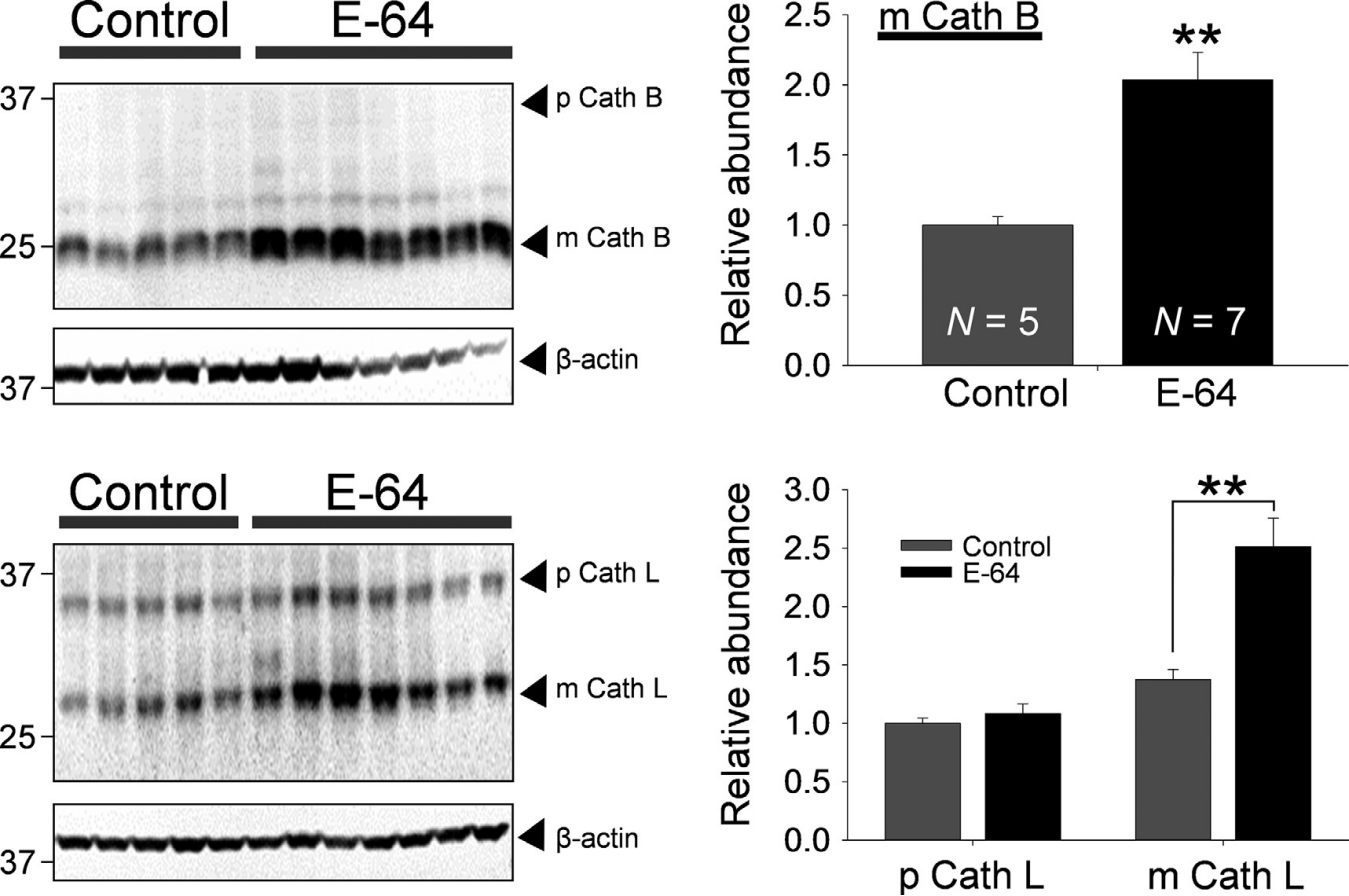
E‐64 treated rats had increased mature Cath B and L levels in the renal cortex. Western blot analysis of (A) Cath B and (B) Cath L protein expression in renal cortex tissue homogenates prepared from E‐64 treated and control rats fed a 8.0% NaCl diet for 21 days. Mature (m) Cath B was detected at 25 kDa but no pro (p) Cath B was detected at the reported 37 kDa. Pro (p) Cath L was detected at 28 kDa and mature (m) Cath L at 37 kDa. Equal loading was verified by anti‐*β*‐actin antibodies. The summary graphs (right panels) show the densitometric analysis of relative Cath abundance. **Significant difference between E‐64 treated and control salt‐sensitive rats, *P *<* *0.01 (Control *N* = 5, E‐64 *N* = 7; error bars, SE).

Previously, inhibition of cysteine Caths by E‐64 has been shown to increase the half‐lives of cysteine Caths due to the requirement of functional cysteine Caths for their degradation (Kominami et al. 1987; Katunuma 2010). This provides a useful tool to assess whether in vivo administration E‐64 was effective at inhibiting Caths in the renal tissue. Specifically, Cath B and L kidney abundance was measured as they are two primary targets of E‐64 (Hashida et al. 1982). A significant increase in the renal cortical mature form of Cath B (Fig. 7A) and Cath L (Fig. 7B) were measured in E‐64 treated rats (*P *<* *0.01, independent‐sample *t*‐test). The pro Cath L abundance was not different between the groups and pro Cath B was not detected.

## Discussion

The goal of this study was to identify whether cysteine Caths modulate the development of SS hypertension and kidney injury in vivo. Multiple studies previously identified that cysteine Caths play a role in the pathogenesis of kidney diseases mostly by their proteolytic activities. For instance, as summarized by Kistler et al. (2010), many glomerular diseases can be regarded as podocyte enzymatic disorders. In addition to the cysteine Cath function in glomeruli, they have been shown to be involved in tubular transport and other processes in the kidney. However, the role of cysteine Caths in the development of hypertension in vivo remains to be determined. We provide evidence here that both the increased MAP and the corresponding kidney damage observed in Dahl SS rats fed a HS diet were not significantly affected by the long‐term E‐64 treatment. Despite the absence of an effect of Cath inhibition on the progression of HS‐induced hypertension, the inhibitor dosage used was sufficient to increase the renal cortical level of mature Cath B and L. As has been previously reported, the increased mature Cath levels suggest that the E‐64 inhibition of Caths impairs their autodegradation. Therefore, E‐64 irreversibly inhibited renal cysteine Caths, but this did not induce any measured changes in the expected pathophysiology of SS hypertension.

Cytoplasmic Cath L has been shown to be required for the induction of proteinuria in mice. Its role is to cleave intracellular proteins involved in the regulation of the podocyte actin cytoskeleton (Reiser et al. 2004; Sever et al. 2007; Faul et al. 2008). Podocytes will then retract their foot processes that may result in podocyte effacement. However, our data revealed that Cath inhibition by the studied E‐64 dose in this particular model had no effect on the HS‐induced podocyte dysfunction and albuminuria. Recently, Cath D has been shown to be important in this process (Yamamoto‐Nonaka et al. 2016). Cath D belongs to the aspartyl group of Caths. Thus, E‐64, used in this study, would not have inhibited Cath D and its function in SS hypertension proteinuria. Interestingly, Cath B and L have been shown to be involved in the regulation of Cath D (Wille et al. 2004; Laurent‐Matha et al. 2006; Zheng et al. 2008), which might explain this inconsistency between our and the previously reported data.

The role of cysteine Caths in auto activation and degradation, as is present in most other Caths, is well known (Kominami et al. 1987; Katunuma 2010). Previously, Caths were viewed as redundant but newer evidence has revealed that each type has specific functions as well as some being subcellularly isolated from each other. The increased mature Cath L in the E‐64 treated group but not in the proform supports that E‐64 causes increased half‐lives of mature Caths by impairing their autodegradation and not their increased activation or expression of Caths. However, the increased protein level of Cath B and Cath L does not indicate the extent of E‐64 inhibition of either Caths. While the E‐64 inhibition of Cath B and L are likely based on E‐64's Cath affinities and the characteristic Cath autodegradation dysfunction, impaired Cath degradation does not indicate that Cath B and L were the primary targets of E‐64. Alternative proteases that are currently unknown to be involved in Cath B and L degradation might have been more inhibited by E‐64. Therefore, an increased catalytic activity is a possible interpretation of the increased renal Cath B and L protein level.

The proteinuria we observed in the SS rats fed a HS diet was expected to be attenuated by E‐64 treatment. In cultured podocytes, E‐64 was recently reported to block an Ang II‐induced, STAT3 mediated loss of the cytoskeleton regulatory protein synaptopodin (Abkhezr and Dryer 2015). The absence of attenuated albuminuria suggests that cysteine Caths are not involved in the development of SS hypertension‐associated proteinuria. Furthermore, the basal podocyte [Ca^2+^]_i_ was similar to previously reported levels (Ilatovskaya et al. 2015a).

Another potential mechanism, which might modulate the development of SS hypertension by cysteine Caths, is the control of the epithelial Na^+^ channel (ENaC)‐mediated sodium absorption in the collecting ducts. ENaC is the main sodium transporter in the aldosterone‐sensitive distal nephron (Staruschenko 2012) and its activity significantly contributes to the development of salt‐induced hypertension in Dahl SS rats (Aoi et al. 2006; Kakizoe et al. 2009; Pavlov et al. 2013). It is well established that activity of ENaC is enhanced by proteolytic cleavage (Bruns et al. 2007; Svenningsen et al. 2009; Ray and Kleyman 2015; Zachar et al. 2015). It was also reported that the cysteine protease Cath S can activate ENaC which may be relevant under pathophysiological conditions (Haerteis et al. 2012). Similarly, other Caths, such as Caths B, H, and S, were shown to modulate ENaC activity in both kidneys and lungs (Alli et al. 2012; Tan et al. 2014; Evans et al. 2016). Considering that Caths are precisely modulated under physiological and pathophysiological conditions, as was shown for Cath D that is significantly altered in response to the activation of the vasopressin signaling (Pisitkun et al. 2006), E‐64 may also be expected to have some effects on the development of SS hypertension via its effects on ENaC activity. However, changes in electrolyte excretion would have been expected but was not observed in the E‐64‐treated animals.

We have shown here evidence that E‐64 does not affect either the blood pressure control or kidney injury in Dahl SS rats fed a HS diet. This might indicate that cysteine Caths are not involved in the progression of SS hypertension. Alternatively, other Caths and cellular proteases may be involved in SS hypertension and kidney damage. Our findings indicate that in vivo inhibition of cysteine Caths may be an ineffective method of attenuating SS hypertension as was indicated by the increased renal Cath B and L protein levels and the lack of attenuated pathophysiology. Likewise, the interregulation of Caths may offset any beneficial inhibitory affects. Future studies using more selective Cath inhibitors may be needed to identify in vivo Cath functions in SS hypertension.

## Conflict of Interest

There are no conflicts of interests.

## References

[phy212950-bib-0001] Abkhezr, M. , and S. E. Dryer . 2015 STAT3 regulates steady‐state expression of synaptopodin in cultured mouse podocytes. Mol. Pharmacol. 87:231–239. 25425624 10.1124/mol.114.094508

[phy212950-bib-0002] Alcalay, N. I. , M. Sharma , D. Vassmer , B. Chapman , B. Paul , J. Zhou , et al. 2008 Acceleration of polycystic kidney disease progression in cpk mice carrying a deletion in the homeodomain protein Cux1. Am. J. Physiol. Renal. Physiol. 295:F1725–F1734. 18829740 10.1152/ajprenal.90420.2008 PMC2604819

[phy212950-bib-0003] Alli, A. A. , J. Z. Song , O. Al‐Khalili , H.‐F. Bao , H.‐P. Ma , A. A. Alli , et al. 2012 Cathepsin B is secreted apically from Xenopus 2F3 cells and cleaves the epithelial sodium channel (ENaC) to increase its activity. J. Biol. Chem. 287:30073–30083. 22782900 10.1074/jbc.M111.338574 PMC3436264

[phy212950-bib-0004] Aoi, W. , N. Niisato , Y. Sawabe , H. Miyazaki , and Y. Marunaka . 2006 Aldosterone‐induced abnormal regulation of ENaC and SGK1 in Dahl salt‐sensitive rat. Biochem. Biophys. Res. Commun. 341:376–381. 16426574 10.1016/j.bbrc.2005.12.194

[phy212950-bib-0005] Bauer, Y. , P. Hess , C. Qiu , A. Klenk , B. Renault , D. Wanner , et al. 2011 Identification of cathepsin L as a potential sex‐specific biomarker for renal damage. Hypertension 57:795–801. 21357272 10.1161/HYPERTENSIONAHA.110.157206

[phy212950-bib-0006] Bruns, J. B. , M. D. Carattino , S. Sheng , A. B. Maarouf , O. A. Weisz , J. M. Pilewski , et al. 2007 Epithelial Na+ channels are fully activated by furin‐ and prostasin‐dependent release of an inhibitory peptide from the gamma‐subunit. J. Biol. Chem. 282:6153–6160. 17199078 10.1074/jbc.M610636200

[phy212950-bib-0007] Cheng, X. W. , T. Murohara , M. Kuzuya , H. Izawa , T. Sasaki , K. Obata , et al. 2008 Superoxide‐dependent cathepsin activation is associated with hypertensive myocardial remodeling and represents a target for angiotensin II type 1 receptor blocker treatment. Am. J. Pathol. 173:358–369. 18583318 10.2353/ajpath.2008.071126 PMC2475774

[phy212950-bib-0008] Cowley, A. W., Jr. , C. Yang , V. Kumar , J. Lazar , H. Jacob , A. M. Geurts , et al. 2016 Pappa2 is linked to salt‐sensitive hypertension in Dahl S rats. Physiol. Genomics 48:62–72. 26534937 10.1152/physiolgenomics.00097.2015 PMC4757026

[phy212950-bib-0009] Endres, B. T. , J. R. Priestley , O. Palygin , M. J. Flister , M. J. Hoffman , B. D. Weinberg , et al. 2014 Mutation of Plekha7 attenuates salt‐sensitive hypertension in the rat. Proc. Natl Acad. Sci. USA 111:12817–12822. 25136115 10.1073/pnas.1410745111 PMC4156702

[phy212950-bib-0010] Evans, T. I. , N. S. Joo , N. W. Keiser , Z. Yan , S. R. Tyler , W. Xie , et al. 2016 Glandular proteome identifies antiprotease cystatin C as a critical modulator of airway hydration and clearance. Am. J. Respir. Cell Mol. Biol. 54:469–481. 26334941 10.1165/rcmb.2015-0090OC PMC4821051

[phy212950-bib-0011] Faul, C. , M. Donnelly , S. Merscher‐Gomez , Y. H. Chang , S. Franz , J. Delfgaauw , et al. 2008 The actin cytoskeleton of kidney podocytes is a direct target of the antiproteinuric effect of cyclosporine A. Nat. Med. 14:931–938. 18724379 10.1038/nm.1857 PMC4109287

[phy212950-bib-0012] Fox, C. , P. Cocchiaro , F. Oakley , R. Howarth , K. Callaghan , J. Leslie , et al. 2016 Inhibition of lysosomal protease cathepsin D reduces renal fibrosis in murine chronic kidney disease. Sci. Rep. 6:20101. 26831567 10.1038/srep20101 PMC4735715

[phy212950-bib-0013] Goulet, B. , A. Baruch , N. S. Moon , M. Poirier , L. L. Sansregret , A. Erickson , et al. 2004 A cathepsin L isoform that is devoid of a signal peptide localizes to the nucleus in S phase and processes the CDP/Cux transcription factor. Mol. Cell 14:207–219. 15099520 10.1016/s1097-2765(04)00209-6

[phy212950-bib-0014] Haerteis, S. , M. Krappitz , M. Bertog , A. Krappitz , V. Baraznenok , I. Henderson , et al. 2012 Proteolytic activation of the epithelial sodium channel (ENaC) by the cysteine protease cathepsin‐S. Pflügers Arch. Eur. J. Physiol. 464:353–365. 22864553 10.1007/s00424-012-1138-3 PMC3448907

[phy212950-bib-0015] Hashida, S. , E. Kominami , and N. Katunuma . 1982 Inhibitions of cathepsin B and cathepsin L by E‐64 in vivo. II. Incorporation of [3H]E‐64 into rat liver lysosomes in vivo. J. Biochem. 91:1373–1380. 7096292 10.1093/oxfordjournals.jbchem.a133825

[phy212950-bib-0016] Igarashi, P. , and S. Somlo . 2007 Polycystic kidney disease. J. Am. Soc. Nephrol. 18:1371–1373. 17429047 10.1681/ASN.2007030299

[phy212950-bib-0017] Ilatovskaya, D. , and A. Staruschenko . 2013 Single‐channel analysis of TRPC channels in the podocytes of freshly isolated glomeruli. Methods Mol. Biol. 998:355–369. 23529444 10.1007/978-1-62703-351-0_28 PMC4181531

[phy212950-bib-0018] Ilatovskaya, D. V. , O. Palygin , V. Chubinskiy‐Nadezhdin , Y. A. Negulyaev , R. Ma , L. Birnbaumer , et al. 2014 Angiotensin II has acute effects on TRPC6 channels in podocytes of freshly isolated glomeruli. Kidney Int. 86:506–514. 24646854 10.1038/ki.2014.71 PMC4149864

[phy212950-bib-0019] Ilatovskaya, D. V. , V. Levchenko , A. Lowing , L. S. Shuyskiy , O. Palygin , and A. Staruschenko . 2015a Podocyte injury in diabetic nephropathy: implications of angiotensin II‐dependent activation of TRPC channels. Sci. Rep. 5:17637. 26656101 10.1038/srep17637 PMC4674698

[phy212950-bib-0020] Ilatovskaya, D. V. , O. Palygin , V. Levchenko , and A. Staruschenko . 2015b Single‐channel analysis and calcium imaging in the podocytes of the freshly isolated glomeruli. J. Vis. Exp. 100:e52850. 10.3791/52850 PMC4544950 26167808

[phy212950-bib-0021] Illy, C. , O. Quraishi , J. Wang , E. Purisima , T. Vernet , and J. S. Mort . 1997 Role of the occluding loop in cathepsin B activity. J. Biol. Chem. 272:1197–1202. 8995421 10.1074/jbc.272.2.1197

[phy212950-bib-0022] Jaiswal, J. K. , N. W. Andrews , and S. M. Simon . 2002 Membrane proximal lysosomes are the major vesicles responsible for calcium‐dependent exocytosis in nonsecretory cells. J. Cell Biol. 159:625–635. 12438417 10.1083/jcb.200208154 PMC2173094

[phy212950-bib-0023] Kakizoe, Y. , K. Kitamura , T. Ko , N. Wakida , A. Maekawa , T. Miyoshi , et al. 2009 Aberrant ENaC activation in Dahl salt‐sensitive rats. J. Hypertens. 27:1679–1689. 19458538 10.1097/HJH.0b013e32832c7d23

[phy212950-bib-0024] Karpushev, A. V. , V. Levchenko , D. V. Ilatovskaya , T. S. Pavlov , and A. Staruschenko . 2011 Novel role of Rac1/WAVE signaling mechanism in regulation of the epithelial Na+ channel. Hypertension 57:996–1002. 21464391 10.1161/HYPERTENSIONAHA.110.157784

[phy212950-bib-0025] Katunuma, N. 2010 Posttranslational processing and modification of cathepsins and cystatins. J. Signal Transduct. 2010:375345. 21637353 10.1155/2010/375345 PMC3100116

[phy212950-bib-0026] Kistler, A. D. , V. Peev , A.‐L. Forst , S. El Hindi , M. M. Altintas , and J. Reiser . 2010 Enzymatic disease of the podocyte. Pediatr. Nephrol. 25:1017–1023. 20130922 10.1007/s00467-009-1425-1 PMC4109305

[phy212950-bib-0027] Kominami, E. , T. Tsukahara , Y. Bando , and N. Katunuma . 1985 Distribution of cathepsins B and H in rat tissues and peripheral blood cells. J. Biochem. 98:87–93. 3900059 10.1093/oxfordjournals.jbchem.a135277

[phy212950-bib-0028] Kominami, E. , T. Tsukahara , Y. Bando , and N. Katunuma . 1987 Autodegradation of lysosomal cysteine proteinases. Biochem. Biophys. Res. Commun. 144:749–756. 3555495 10.1016/s0006-291x(87)80028-1

[phy212950-bib-0029] Laurent‐Matha, V. , D. Derocq , C. Prebois , N. Katunuma , and E. Liaudet‐Coopman . 2006 Processing of human cathepsin D is independent of its catalytic function and auto‐activation: involvement of cathepsins L and B. J. Biochem. 139:363–371. 16567401 10.1093/jb/mvj037 PMC2376303

[phy212950-bib-0030] Lavin, P. J. , and M. P. Winn . 2011 TORCing up the importance of calcium signaling. J. Am. Soc. Nephrol. 22:1391–1393. 21757772 10.1681/ASN.2011060595

[phy212950-bib-0031] Moreno, C. , J. M. Williams , L. Lu , M. Liang , J. Lazar , H. J. Jacob , et al. 2011 Narrowing a region on rat chromosome 13 that protects against hypertension in Dahl SS‐13BN congenic strains. Am. J. Physiol. Heart Circ. Physiol. 300:H1530–H1535. 21257920 10.1152/ajpheart.01026.2010 PMC3075031

[phy212950-bib-0032] Muntener, K. , R. Zwicky , G. Csucs , J. Rohrer , and A. Baici . 2004 Exon skipping of cathepsin B: mitochondrial targeting of a lysosomal peptidase provokes cell death. J. Biol. Chem. 279:41012–41017. 15262981 10.1074/jbc.M405333200

[phy212950-bib-0033] Musil, D. , D. Zucic , D. Turk , R. A. Engh , I. Mayr , R. Huber , et al. 1991 The refined 2.15 A X‐ray crystal structure of human liver cathepsin B: the structural basis for its specificity. EMBO J. 10:2321–2330. 1868826 10.1002/j.1460-2075.1991.tb07771.x PMC452927

[phy212950-bib-0034] Nagase, M. , S. Shibata , S. Yoshida , T. Nagase , T. Gotoda , and T. Fujita . 2006 Podocyte injury underlies the glomerulopathy of Dahl salt‐hypertensive rats and is reversed by aldosterone blocker. Hypertension 47:1084–1093. 16636193 10.1161/01.HYP.0000222003.28517.99

[phy212950-bib-0035] Pavenstadt, H. , W. Kriz , and M. Kretzler . 2003 Cell biology of the glomerular podocyte. Physiol. Rev. 83:253–307. 12506131 10.1152/physrev.00020.2002

[phy212950-bib-0036] Pavlov, T. S. , V. Levchenko , P. M. O'Connor , D. V. Ilatovskaya , O. Palygin , T. Mori , et al. 2013 Deficiency of renal cortical EGF increases ENaC activity and contributes to salt‐sensitive hypertension. J. Am. Soc. Nephrol. 24:1053–1062. 23599382 10.1681/ASN.2012080839 PMC3699826

[phy212950-bib-0037] Pisitkun, T. , J. Bieniek , D. Tchapyjnikov , G. Wang , W. W. Wu , R.‐F. Shen , et al. 2006 High‐throughput identification of IMCD proteins using LC‐MS/MS. Physiol. Genomics 25:263–276. 16449382 10.1152/physiolgenomics.00214.2005 PMC1436036

[phy212950-bib-0038] Ray, E. C. , and T. R. Kleyman . 2015 Cutting it out: ENaC processing in the human nephron. J. Am. Soc. Nephrol. 26:1–3. 25060054 10.1681/ASN.2014060618 PMC4279749

[phy212950-bib-0039] Reddy, A. , E. V. Caler , and N. W. Andrews . 2001 Plasma membrane repair is mediated by Ca(2+)‐regulated exocytosis of lysosomes. Cell 106:157–169. 11511344 10.1016/s0092-8674(01)00421-4

[phy212950-bib-0040] Reiser, J. , J. Oh , I. Shirato , K. Asanuma , A. Hug , T. M. Mundel , et al. 2004 Podocyte migration during nephrotic syndrome requires a coordinated interplay between cathepsin L and alpha3 integrin. J. Biol. Chem. 279:34827–34832. 15197181 10.1074/jbc.M401973200

[phy212950-bib-0041] Rossi, A. , Q. Deveraux , B. Turk , and A. Sali . 2004 Comprehensive search for cysteine cathepsins in the human genome. Biol. Chem. 385:363–372. 15195995 10.1515/BC.2004.040

[phy212950-bib-0042] Sever, S. , M. M. Altintas , S. R. Nankoe , C. C. Moller , D. Ko , C. Wei , et al. 2007 Proteolytic processing of dynamin by cytoplasmic cathepsin L is a mechanism for proteinuric kidney disease. J. Clin. Invest. 117:2095–2104. 17671649 10.1172/JCI32022 PMC1934589

[phy212950-bib-0043] Siklos, M. , M. Benaissa , and G. R. Thatcher . 2015 Cysteine proteases as therapeutic targets: does selectivity matter? A systematic review of calpain and cathepsin inhibitors. Acta Pharm. Sin. B 5:506–519. 26713267 10.1016/j.apsb.2015.08.001 PMC4675809

[phy212950-bib-0044] Staruschenko, A. 2012 Regulation of transport in the connecting tubule and cortical collecting duct. Compr. Physiol. 2:1541–1584. 23227301 10.1002/cphy.c110052 PMC3516049

[phy212950-bib-0045] Svenningsen, P. , C. Bistrup , U. G. Friis , M. Bertog , S. Haerteis , B. Krueger , et al. 2009 Plasmin in nephrotic urine activates the epithelial sodium channel. J. Am. Soc. Nephrol. 20:299–310. 19073825 10.1681/ASN.2008040364 PMC2637049

[phy212950-bib-0046] Tan, C. D. , C. Hobbs , M. Sameni , B. F. Sloane , M. J. Stutts , and R. Tarran . 2014 Cathepsin B contributes to Na+ hyperabsorption in cystic fibrosis airway epithelial cultures. J. Physiol. 592:5251–5268. 25260629 10.1113/jphysiol.2013.267286 PMC4262337

[phy212950-bib-0047] Turk, V. , V. Stoka , O. Vasiljeva , M. Renko , T. Sun , B. Turk , et al. 2012 Cysteine cathepsins: from structure, function and regulation to new frontiers. Biochim. Biophys. Acta 1824:68–88. 22024571 10.1016/j.bbapap.2011.10.002 PMC7105208

[phy212950-bib-0048] Wille, A. , A. Gerber , A. Heimburg , A. Reisenauer , C. Peters , P. Saftig , et al. 2004 Cathepsin L is involved in cathepsin D processing and regulation of apoptosis in A549 human lung epithelial cells. Biol. Chem. 385:665–670. 15318816 10.1515/BC.2004.082

[phy212950-bib-0049] Yamamoto‐Nonaka, K. , M. Koike , K. Asanuma , M. Takagi , J. A. Oliva Trejo , T. Seki , et al. 2016 Cathepsin D in podocytes is important in the pathogenesis of proteinuria and CKD. J. Am. Soc. Nephrol. 27:1–16. 26823550 10.1681/ASN.2015040366 PMC5004641

[phy212950-bib-0050] Zachar, R. M. , K. Skjødt , N. Marcussen , S. Walter , A. Toft , M. R. Nielsen , et al. 2015 The epithelial sodium channel *γ*‐subunit is processed proteolytically in human kidney. J. Am. Soc. Nephrol. 26:95–106. 25060057 10.1681/ASN.2013111173 PMC4279735

[phy212950-bib-0051] Zheng, X. , F. Chu , B. L. Mirkin , T. Sudha , S. A. Mousa , and A. Rebbaa . 2008 Role of the proteolytic hierarchy between cathepsin L, cathepsin D and caspase‐3 in regulation of cellular susceptibility to apoptosis and autophagy. Biochim. Biophys. Acta 1783:2294–2300. 18775751 10.1016/j.bbamcr.2008.07.027

